# Pancreatic fatty replacement as risk marker for altered glucose metabolism and cardiac iron and complications in thalassemia major

**DOI:** 10.1007/s00330-023-09630-z

**Published:** 2023-04-28

**Authors:** Antonella Meloni, Mario Nobile, Petra Keilberg, Vincenzo Positano, Maria Filomena Santarelli, Laura Pistoia, Anna Spasiano, Tommaso Casini, Maria Caterina Putti, Liana Cuccia, Pier Paolo Bitti, Giuseppe Messina, Giuseppe Peritore, Stefania Renne, Emanuele Grassedonio, Emilio Quaia, Filippo Cademartiri, Alessia Pepe

**Affiliations:** 1 Department of Radiology, Fondazione G. Monasterio CNR-Regione Toscana, Pisa, Italy; 2 U. O. C. Bioingegneria, Fondazione G. Monasterio CNR-Regione Toscana, Pisa, Italy; 3 Sezione Di Scienze Radiologiche - Dipartimento Di Biopatologia E Biotecnologie Mediche, Policlinico “Paolo Giaccone”, Palermo, Italy; 4 Istituto Di Fisiologia Clinica, Consiglio Nazionale Delle Ricerche, Pisa, Italy; 5 Unità Operativa Semplice Dipartimentale Malattie Rare del Globulo Rosso, Azienda Ospedaliera Di Rilievo Nazionale “A. Cardarelli”, Napoli, Italy; 6 Centro Talassemie Ed Emoglobinopatie, Ospedale “Meyer”, Florence, Italy; 7 Dipartimento Della Salute Della Donna E del Bambino, Clinica Di Emato-Oncologia Pediatrica, Azienda Ospedaliero-Università Di Padova, Padua, Italy; 8 Unità Operativa Complessa Ematologia Con Talassemia, ARNAS Civico “Benfratelli-Di Cristina”, Palermo, Italy; 9 Dipartimento Dei Servizi, Servizio Immunoematologia E Medicina Trasfusionale, Presidio Ospedaliero “San Francesco” ASL Nuoro, Nuoro, Italy; 10 Centro Microcitemie, Grande Ospedale Metropolitano “Bianchi-Melacrino-Morelli”, Reggio Calabria, Italy; 11 Unità Operativa Complessa Di Radiologia, ARNAS Civico “Benfratelli-Di Cristina”, Palermo, Italy; 12 Struttura Complessa Di Cardioradiologia-UTIC, Presidio Ospedaliero “Giovanni Paolo II”, Lamezia Terme, Italy; 13 Institute of Radiology, Department of Medicine, University of Padua, Giustiniani, 2 Street, 35128 Padova, Italy

**Keywords:** Pancreas, Magnetic resonance imaging, Thalassemia

## Abstract

**Objectives:**

This multicenter study assessed the extent of pancreatic fatty replacement and its correlation with demographics, iron overload, glucose metabolism, and cardiac complications in a cohort of well-treated patients with thalassemia major (TM).

**Methods:**

We considered 308 TM patients (median age: 39.79 years; 182 females) consecutively enrolled in the Extension-Myocardial Iron Overload in Thalassemia Network. Magnetic resonance imaging was used to quantify iron overload (IO) and pancreatic fat fraction (FF) by T2* technique, cardiac function by cine images, and to detect replacement myocardial fibrosis by late gadolinium enhancement technique. The glucose metabolism was assessed by the oral glucose tolerance test.

**Results:**

Pancreatic FF was associated with age, body mass index, and history of hepatitis C virus infection. Patients with normal glucose metabolism showed a significantly lower pancreatic FF than patients with impaired fasting glucose (*p* = 0.030), impaired glucose tolerance (*p* < 0.0001), and diabetes (*p* < 0.0001). A normal pancreatic FF (< 6.6%) showed a negative predictive value of 100% for abnormal glucose metabolism. A pancreatic FF > 15.33% predicted the presence of abnormal glucose metabolism. Pancreas FF was inversely correlated with global pancreas and heart T2* values. A normal pancreatic FF showed a negative predictive value of 100% for cardiac iron. Pancreatic FF was significantly higher in patients with myocardial fibrosis (*p* = 0.002). All patients with cardiac complications had fatty replacement, and they showed a significantly higher pancreatic FF than complications-free patients (*p* = 0.002).

**Conclusion:**

Pancreatic FF is a risk marker not only for alterations of glucose metabolism, but also for cardiac iron and complications, further supporting the close link between pancreatic and cardiac disease.

**Key Points:**

• In thalassemia major, pancreatic fatty replacement by MRI is a frequent clinical entity, predicted by a pancreas T2* < 20.81 ms and associated with a higher risk of alterations in glucose metabolism.

*• In thalassemia major, pancreatic fatty replacement is a strong risk marker for cardiac iron, replacement fibrosis, and complications, highlighting a deep connection between pancreatic and cardiac impairment.*

## Introduction

The introduction of oral iron chelation therapy and of the T2* magnetic resonance imaging (MRI) technique for the non-invasive quantification of myocardial iron overload (MIO) has significantly increased the life expectancy of patients with thalassemia major (TM), needing lifelong regular blood transfusions [1–3]. With an aging TM population, diabetes mellitus (DM) is an increasing issue. Age per se is one of the most important risk factors in the development of hyperglycemia, leading to both deficiency of insulin secretion and insulin resistance [4]. In TM, these processes are aggravated/accelerated by pancreatic [5] and hepatic [6] IO. In TM, the prevalence of pancreatic iron is close to 90% and a normal global pancreas T2* value was found to have a negative predictive value of 100% for disturbances of glucose metabolism [7, 8]. However, pancreatic iron has a low specificity for glucose dysregulation [8], which depends on IO severity and duration.

It has been hypothesized that, after the death of the pancreatic cells caused by the cytotoxic iron effect, a progressive fatty replacement of the pancreatic parenchyma may occur [9]. MRI represents the best imaging technique for evaluating the deposition of ectopic fat [10, 11]. However, pancreatic fatty replacement and its clinical correlations in TM have been little explored. Midiri et al [9] measured the pancreas-to-fat signal intensity ratio (SIR) in T1-weighted, T2-weighted, and T2*-weighted sequences in 20 TM patients and noticed an increased SIR, attributed to a progressive substitution of the parenchyma by inert adipose tissue, in 3 (15%) patients. Papakonstantinou et al [12] used the signal intensity change between in-phase (water + fat) and opposed-phase (water - fat) images, named pancreatic signal index (PSI), as an index for pancreatic fat. A PSI > 20% was considered indicative of pancreatic fatty replacement, diagnosed in 45% of the 31 included TM patients. The fatty replacement was more frequent in diabetic than in non-diabetic patients (77% vs 20%) [12]. However, a negative PSI was detected in 9 patients, due to the predominant effect of iron deposition. Indeed, the ability of this technique to quantify fat is corrupted by multiple confounding factors, especially in the presence of low-fat fractions (FF) and high iron levels [13]. Huang et al [14] measured pancreatic FF by using the iterative decomposition of water and fat with echo asymmetry and the least-squares estimation algorithm in 40 pediatric TM patients. Pancreatic FF was associated with pancreatic iron and altered glucidic metabolism.

One attractive approach to quantify the FF by MRI is to take advantage of the conventional gradient-echo multiecho T2* images used for iron quantification. The pancreas T2* is obtained from these images by fitting the signal to an appropriate decay model, and the fat generates a sinusoidal signal fluctuation over-imposed to the exponential decay [15]. An appropriate fitting model can be used to separate the fat signal from the water contribution [16, 17]. This approach, introduced and largely used for the assessment of hepatic FF, has been extended to the pancreas [18, 19]. In a cohort of 71 patients with different iron overload diseases, pancreatic FF was found associated with pancreatic iron and exocrine function [18].

To the best of our knowledge, no data are available about the association between pancreatic FF and MIO in TM patients. Moreover, since pancreatic steatosis may have a causative effect and contribute to the development of diabetes and diabetes was shown to increase the risk for heart failure (HF), hyperkinetic arrhythmias, and myocardial fibrosis independently of MIO [20], a profound link between pancreatic fat and heart disease could be foreseen.

This multicenter study aimed to assess the extent of pancreatic fatty replacement and its correlation with demographics, iron overload, glucose metabolism, and cardiac complications in a cohort of well-treated TM patients.

## Materials and methods

### Study population

We considered 308 β-TM patients (182 females, median age: 39.79 years) consecutively enrolled in the Extension-Myocardial Iron Overload in Thalassemia (E-MIOT) project. The E-MIOT is an Italian network constituted of 66 thalassemia centers and 11 MRI sites, where MRI exams are performed using homogeneous, standardized, and validated procedures [21–23]. All centers are linked by a shared database, collecting all clinical, laboratory, and instrumental data.

Moreover, with the aim of defining the upper limit of pancreatic FF, we included 20 healthy subjects (10 females, median age: 35.76 years) without pancreatic diseases, alterations of glucidic metabolism, or known conditions/treatments which could affect pancreatic iron content or fat.

The study complied with the Declaration of Helsinki and was approved by the ethical committees of all the MRI sites. All subjects gave written informed consent.

### MRI

All patients underwent MRI using conventional clinical 1.5 T scanners from three main vendors.

Five or more axial slices including the whole pancreas [24], a mid-transverse hepatic slice [25], and basal, medium, and apical short-axis views of the left ventricle (LV) [21, 26] were acquired with T2* multiecho gradient-echo sequences. T2* image analysis was performed using a custom-written, previously validated software (HIPPO MIOT®) [27]. Three regions of interest (ROIs) were manually drawn over the pancreatic head, body, and tail, encompassing parenchymal tissue and avoiding large blood vessels or ducts [15]. For each ROI, the mean value of the signal intensity along all echo times was calculated. Each obtained decay curve was fit to a multipeak fat model in order to estimate both T2* and FF [16, 18, 19]. Global pancreatic FF/T2* values were evaluated as the mean of FF/T2* values from the three regions. Hepatic T2* values were calculated in a circular ROI [28] and converted into liver iron concentration (LIC) [29, 30]. The myocardial T2* distribution was mapped into a 16-segment LV model, according to the American Heart Association standardized segmentation (six equiangular segments in the basal and medium slices and four in the apical slice) [31]. For each segment, the mean value of the signal intensity along all the echo times was calculated and the assessed decay curve was fit to the single exponential model. In heavily iron-overloaded hearts, a truncation model was applied to delete the late points with a low signal-to-noise ratio [32]. An appropriate correction map was applied to correct for susceptibility artifacts [27]. The global heart T2* value was the mean of all segmental values.

Steady-state free precession cine images were acquired in sequential 8-mm short-axis slices from the atrio-ventricular ring to the apex to quantify biventricular function parameters in a standard [33] and reproducible [34] way. The analysis was based on the manual recognition of the endocardial and epicardial borders of the wall, at least in the end-diastolic and end-systolic phases in each slice. The papillary muscles were delineated and were considered myocardial mass rather than part of the blood pool. For the calculation of end-diastolic and end-systolic volumes (EDV and ESV, respectively), no geometric assumption of the ventricle shape was needed. The stroke volume index (SVI) was calculated as the difference between the EDV index (EDVI) and ESV index (ESVI). The ejection fraction (EF) was given by the ratio between the SVI and the EDVI [33].

Short-axis and long-axis late gadolinium enhancement (LGE) images were acquired by a fast gradient-echo inversion recovery sequence 10–18 min after gadobutrol (Gadovist®; Bayer) intravenous administration (0.2 mmol/kg) to detect replacement myocardial fibrosis [35, 36]. LGE images were not acquired in patients with renal problems or refusing the contrast medium. LGE was considered present when visualized in two different views [37].

### Assessment of glucose metabolism

To assess the disturbances of glucose metabolism, patients not already diagnosed with diabetes performed an oral glucose tolerance test (OGTT) within 3 months from the MRI study at the reference thalassemia center.

Baseline (after overnight fasting) blood assessments of glucose and insulin were performed. Patients were given 1.75 g/kg (maximum dose = 75 g) of glucose solution; glucose and insulin were measured at 60 and 120 min. In patients without known diabetes, we used the homeostasis model assessment of insulin resistance (HOMA-IR) index to assess the insulin resistance [HOMA-IR = (glucose X insulin)/405] [38].

### Diagnostic criteria

The upper limit of pancreatic FF in healthy individuals was defined on log-transformed data as mean + 2standard deviations (SD).

The lowest threshold of normal T2* pancreatic value was 26 ms [24]. A LIC ≥ 3 mg/g/dw indicated significant hepatic iron load [39]. A T2* measurement > 20 ms was taken as a “conservative” normal value for segmental and global heart T2* values [27, 40].

A fasting plasma glucose (FPG) < 100 mg/dL and 2-h glucose < 140 mg/dL were considered normal glucose tolerance (NGT). Impaired fasting glucose (IFG) was diagnosed in presence of FPG levels between 100 and 126 mg/dL. Impaired glucose tolerance (IGT) was defined by 2-h plasma glucose between 140 and 200 mg/dL, with a FPG < 126 mg/dL. DM was defined by FPG ≥ 126 mg/dL or 2-h glucose ≥ 200 mg/dL during an OGTT or random plasma glucose ≥ 200 mg/dL with classic symptoms of hyperglycemia [41].

The metabolic syndrome was defined by the presence of at least 3 of the following criteria: (1) waist circumference ≥ 102 cm in men or ≥ 88 cm in women; (2) high-density lipoprotein < 40 mg/dL in men and < 50 mg/dL in women or on drug treatment; (3) triglycerides ≥ 150 mg/dL or on drug treatment; (4) systolic blood pressure ≥ 130 mmHg or diastolic blood pressure ≥ 85 mmHg or on anti-hypertensive medication; (5) FPG ≥ 100 mg/dL or on treatment for diabetes [42].

HF was diagnosed by clinicians based on symptoms, signs, and instrumental findings according to the AHA/ACC guidelines [43]. Arrhythmias were diagnosed only if ECG-documented and requiring specific medication [44]. Pulmonary hypertension (PH) was diagnosed if the trans-tricuspidal velocity jet was > 3.2 m/s [45]. The term “cardiac complications” included HF, arrhythmias, and PH clinically active at the time of the MRI.

### Statistical analysis

All data were analyzed using the SPSS version.16.0 (SPSS Inc.) and MedCalc version.7.2.1.0 (MedCalc Software) statistical packages.

Due to the non-normal distribution, continuous variables were represented with median and 25th and 75th percentiles. Categorical variables were expressed as frequencies and percentages.

The Friedman test was used to evaluate whether FF was different among the three pancreatic regions. Comparisons between groups were made by Wilcoxon’s signed rank test (for 2 groups) or the Kruskal–Wallis test (for more than 2 groups). The Bonferroni post hoc test was used for multiple comparisons between pairs of groups. Correlation analysis was performed by using Spearman’s test.

The *χ*^2^ test was used for the comparison of non-continuous variables.

To determine the best cutoff for discriminating the presence of a specific condition, the maximum sum of sensitivity and specificity was calculated from receiver-operating characteristic (ROC) curve analysis.

Univariate and stepwise multivariate regression analyses were performed to identify determinants of global pancreas FF. Multivariate regression was performed using only variables with a *p* value < 0.05 in univariate regression analyses. The collinearity of variables tested in the multivariate model was assessed using the variance inflation factor (inflated if > 5) and its tolerance statistic (inflated if < 0.20).

A 2-tailed *p* < 0.05 was considered statistically significant.

## Results

### Pancreatic FF in healthy individuals

The FF was not significantly different among the three pancreatic regions [head: 1.63 (0.00–5.64) %; body: 0.88 (0.00–3.23) %; tail: 1.46 (0.00–4.27) %; *p* = 0.407].

The global pancreatic FF in healthy subjects was 1.85 (1.02–3.26) %, and the upper limit of the pancreatic FF was 6.6%.

The global pancreatic FF was significantly correlated with body mass index (*R* = 0.838; *p* < 0.0001), but it was not associated with age (*R* =  − 0.030; *p* = 0.900).

### Pancreatic FF in TM: distribution and correlation with demographics

The patient’s demographic and clinical characteristics are summarized in Table 1.

**Table 1:** Demographic, clinical, and MRI data of the patients

Variable	Value
Sex (males/females)	126/182
Age (years)	39.79 (31.86–44.71)
Transfusion starting age (years)	1.00 (0.50–2.00)
Chelation starting age (years)	3.00 (2.00–6.00)
Splenectomy, *N* (%)	145 (47.1)
Body mass index (kg/m^2^)	22.27 (20.20–24.61)
HCV infection, *N* (%)	
negative	112/286 (39.2)
eradicated	160/286 (55.9)
positive	14/286 (4.9)
Mean pre-transfusion hemoglobin in the last 12 months (g/dL)	9.70 (9.40–10.00)
Mean serum ferritin in the last 12 months (ng/mL)	700.00 (400.00–1248.00)
Fasting plasma glucose (mg/dL)	90 (81.00–86.00)
1-h plasma glucose after OGTT (mg/dL)	128.50 (107.00–161.00)
2-h plasma glucose after OGTT (mg/dL)	112.00 (96.50–121.00)
HOMA-IR index	1.36 (0.98–2.16)
Glucose metabolism, *N* (%)	
Normal	208/300 (69.3)
Impaired fasting glucose	25/300 (8.3)
Impaired glucose tolerance	19/300 (6.3)
Diabetes mellitus	48/300 (16.0)
Global pancreas T2* (ms)	14.95 (8.82–21.19)
MRI LIC (mg/g dw)	2.41 (1.60–7.59)
Global heart T2* (ms)	39.94 (34.78–43.88)
Left ventricular ejection fraction (%)	63.00 (58.00–67.00)
Right ventricular ejection fraction (%)	61.00 (57.00–66.00)
Myocardial fibrosis, *N* (%)	56/143 (39.2%)

*HCV* hepatitis C virus, *N* number, *FF* fat fraction, *MRI* magnetic resonance imaging, *LIC* liver iron concentration

The FF value was not significantly different among the three pancreatic regions [head: 21.31 (7.96–36.51) %; body: 23.88 (9.25–38.99) %; tail: 21.77 (8.77–37.49) %; *p* = 0.059].

Global pancreatic FF was 24.99 (10.95–38.15) %. Two-hundred and fifty-three (82.1%) patients had a fatty replacement. The youngest patient with pancreatic fatty replacement was 9 years old, and he showed a pathological global pancreas T2*.

The global pancreatic FF was comparable between males and females [23.83 (11.52–38.19) % vs 26.59 (9.96–38.11) %; *p* = 0.411], and it showed a weak positive correlation with age (*R* = 0.324; *p* < 0.0001) and body mass index (*R* = 0.237; *p* < 0.0001).

Prevalence of metabolic syndrome was 4.2% and global pancreatic FF was significantly higher in patients with versus those without metabolic syndrome [34.85 (27.33–48.05) % vs 24.37 (8.29–37.59) %; *p* = 0.021].

Splenectomised patients showed a significantly higher global pancreatic FF value than patients with the spleen [27.49 (16.09–40.55) % vs 23.46 (7.01–35.54) %; *p* = 0.009].

### Pancreatic FF and pancreatic iron overload

**Fig. 1:**
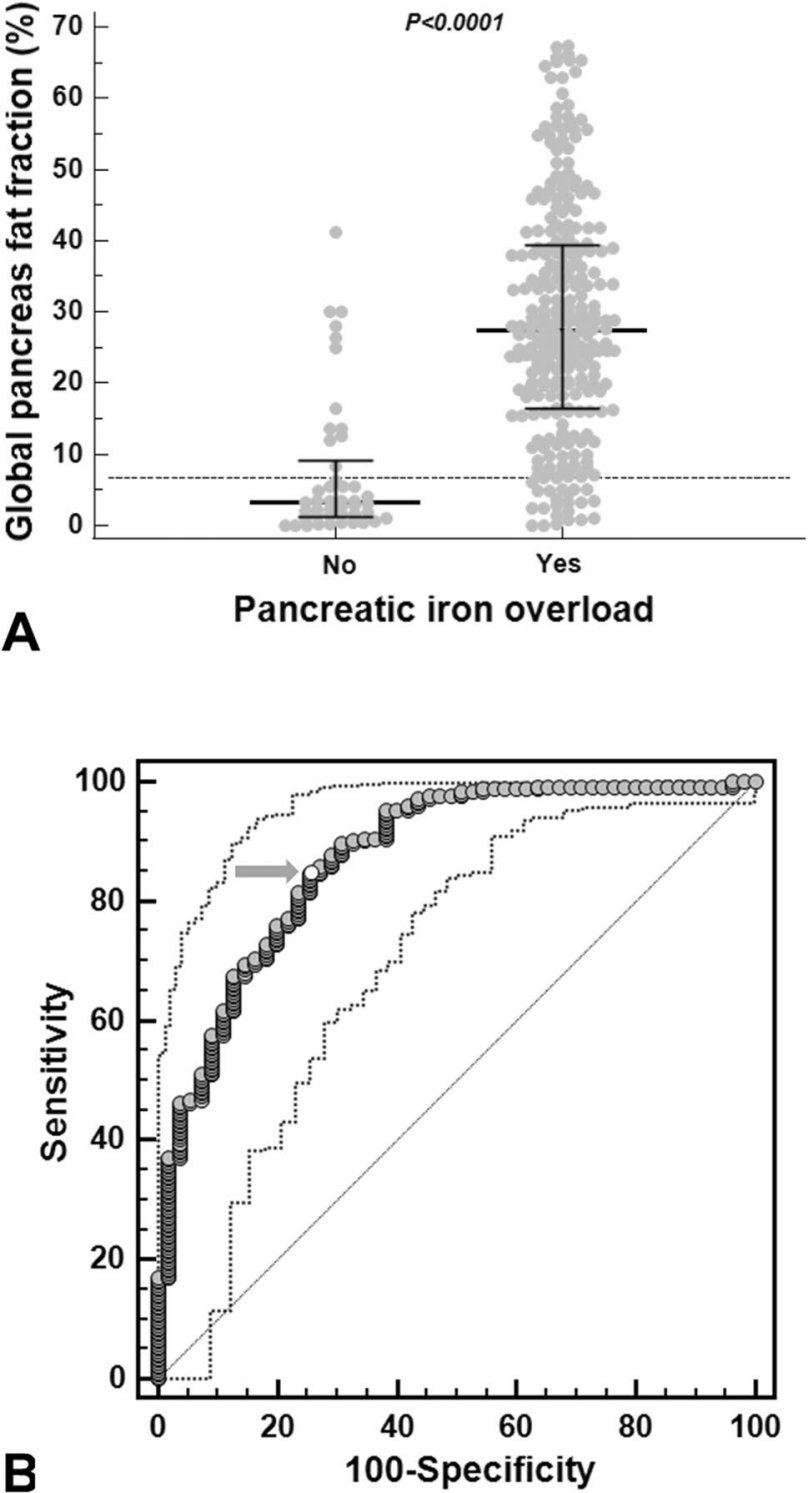
**A** Global pancreas FF in patients without and with pancreatic iron overload. The horizontal dotted line represents the upper limit of normal for global pancreas FF. **B** ROC curve analysis of global pancreas T2* values to predict pancreatic fatty replacement

A significant inverse correlation was detected between global pancreatic FF and T2* values (*R* =  − 0.570; *p* < 0.0001). Two-hundred and sixty-three (85.4%) patients had a global pancreas T2* < 26 ms and they showed a significantly higher global pancreatic FF [27.48 (16.35–39.38) % vs 3.30 (1.35–8.29) %; *p* < 0.0001] (Fig. 1A). The 95.3% of patients with fatty replacement had also pancreatic iron overload. At ROC analysis, a global pancreas T2* < 20.81 ms predicted the presence of fatty replacement with a sensitivity = 84.6% and a specificity = 74.6% (*p* < 0.0001). The area under the curve (AUC) was 0.87 (95% confidence intervals = 0.83–0.91) (Fig. 1B).

### Pancreatic FF and hepatitis C virus (HCV) infection

On the basis of the presence of HCV antibodies and ribonucleic acid, a categorization into three groups was performed: negative patients (group 0; 39.2%), patients who eradicated the virus spontaneously or after treatment with antiviral therapy (group 1; 55.9%), and patients with chronic HCV infection (group 2; 4.9%). Patients in group 0 were significantly younger than patients in group 1 [29.17 (18.85–37.09) years vs 43.26 (39.21–46.46) years; *p* < 0.0001] and in group 2 [29.17 (18.85–37.09) years vs 41.63 (35.79–46.94) years; *p* = 0.045].

Patients in group 0 showed a significantly lower global pancreas FF than patients in group 1 [13.34 (3.51–33.53) % vs 27.98 (18.60–39.32); *p* < 0.0001] and in group 2 [13.34 (3.51–33.53) % vs 28.84 (24.24–40.55) %; *p* = 0.015).

The frequency of diabetes was significantly lower in group 0 versus both group 1 (6.3% vs 20.6%; *p* = 0.003) and group 2 (6.3% vs 21.4%; *p* = 0.048).

### Predictors of global pancreas FF

Past or active HCV infection, serum ferritin levels, body mass index, and pancreatic iron levels were the strongest predictors of global pancreas FF (*F* = 51.68; *p* < 0.0001) (Table 2). No variable was excluded from the multivariable model due to excessive collinearity (tolerance statistic < 0.20 and/or variance inflation factor > 5).

**Table 2:** Univariate and multivariate linear regression analysis for the prediction of the global pancreas FF

	Univariate		Multivariate	
	*β*	*p* value	*β*	*p* value
Female sex	0.036	0.532		
Age	0.329	< 0.0001		
Body mass index	0.233	< 0.0001	0.209	< 0.0001
Metabolic syndrome	0.140	0.024		
Splenectomy	0.146	0.010		
Past or active HCV infection	0.285	< 0.0001	0.207	< 0.0001
Serum ferritin	0.180	0.003	0.097	0.048
MRI LIC	0.098	0.085		
Global pancreas T2*	− 0.579	< 0.0001	− 0.526	< 0.0001

### Pancreatic FF and glucose metabolism

Thirty-nine patients were already diagnosed with diabetes, and 261 were tested for blood glucose. 8.3% of the patients showed IFG, 6.3% IGT, and 16.0% DM.

In non-diabetic patients, the global pancreatic FF showed a weak significant correlation with FPG (*R* = 0.242; *p* < 0.0001), 1-h plasma glucose (*R* = 0.214; *p* = 0.024), and 2-h plasma glucose (*R* = 0.240; *p* = 0.005). No correlation was detected between pancreatic FF and HOMA-IR.

**Fig. 2:**
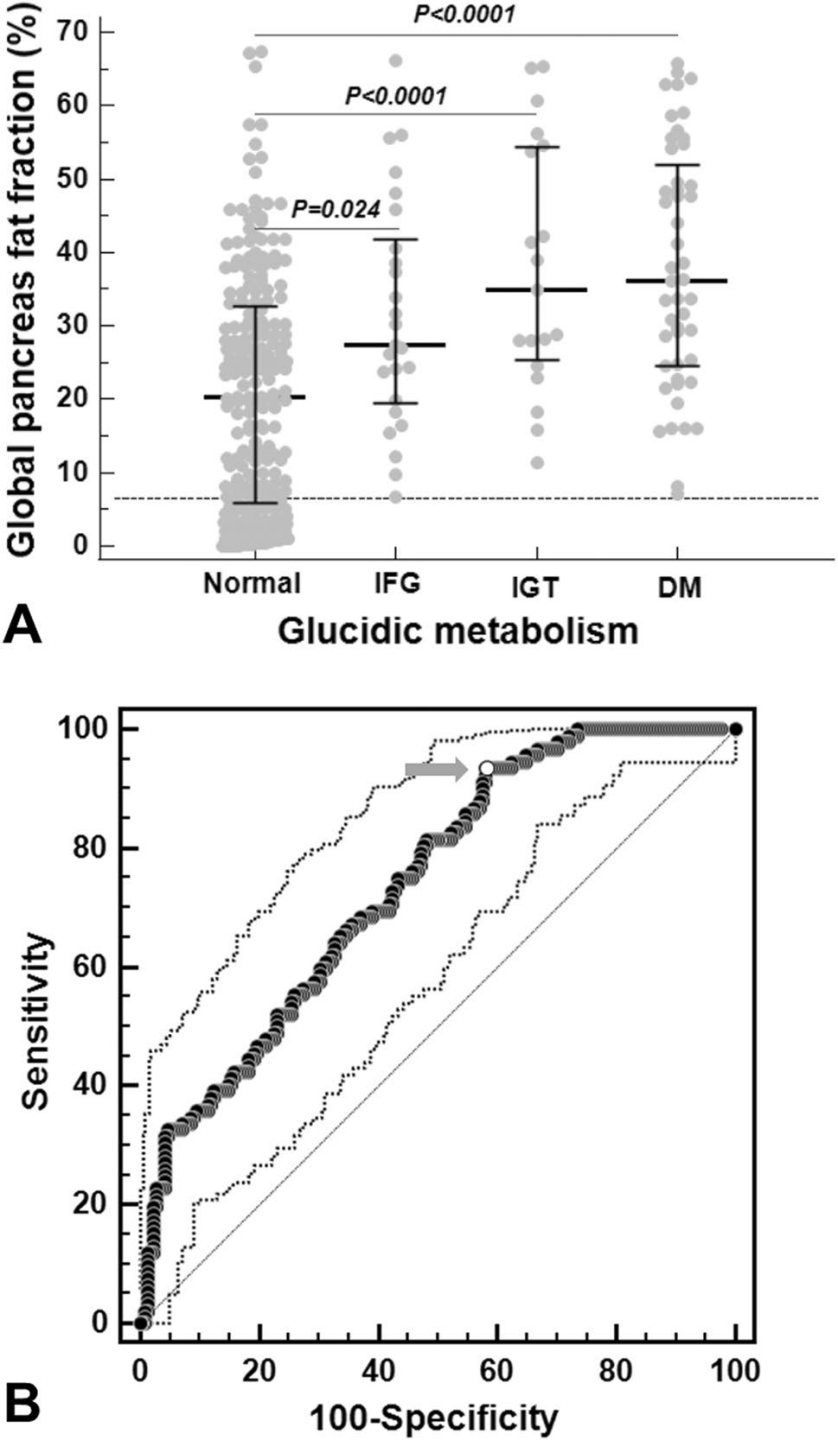
**A** Global pancreas FF in the four groups of patients identified on the basis of the alterations of glucose metabolism. The horizontal dotted line represents the upper limit of normal for global pancreas FF. **B** ROC curve analysis of global pancreas FF to predict the alterations of glucose metabolism

Patients with normal glucose metabolism showed a significantly lower global pancreas FF than patients with IFG [20.27 (5.84–32.60) % vs 27.49 (19.88–40.55) %; *p* = 0.024], IGT [20.27 (5.84–32.60) % vs 34.85 (26.25–54.10) %; *p* < 0.0001], and DM [20.27 (5.84–32.60) % vs 36.18 (24.62–51.85) %; *p* < 0.0001] (Fig. 2A).

The 74.4% of the patients with a normal glucose metabolism had fatty replacement. All patients with altered glucose metabolism had pancreatic fatty replacement and IO. A normal global pancreas FF showed a negative predictive value of 100% for altered glucose metabolism.

At ROC curve analysis, a global pancreas FF > 15.33% predicted the presence of abnormal glucose metabolism with a sensitivity = 93.5% and a specificity = 41.8% (*p* < 0.0001). The AUC was 0.74 (95% confidence interval = 0.69–0.79) (Fig. 2B). A pancreatic T2* < 16.67 ms predicted the presence of abnormal glucose metabolism with a sensitivity = 85.9% and a specificity = 36.1% (*p* < 0.0001). The AUC was 0.65 (95% confidence interval = 0.59–0.70). Delong’s test showed a significant difference among the AUCs (*p* = 0.012).

### Pancreatic FF and iron overload in other organs

No significant correlation was found between global pancreatic FF and LIC values, but the 138 (44.8%) patients with hepatic IO showed a significantly higher global pancreatic FF than patients without hepatic IO [26.95 (15.97–39.42) % vs 23.95 (6.99–34.72) %; *p* = 0.018].

**Fig. 3:**
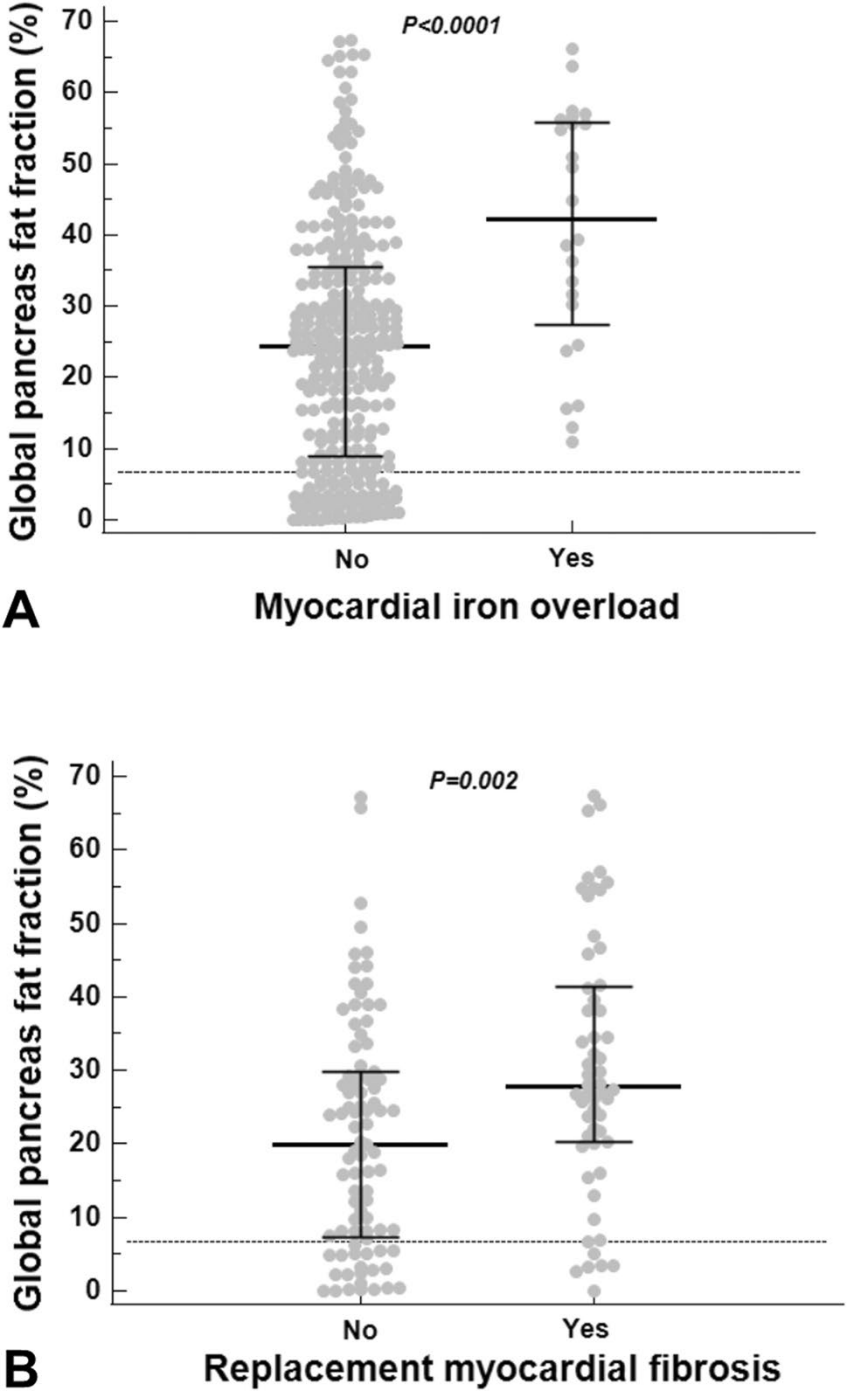
**A** Global pancreas FF in patients without and with myocardial iron overload. **B** Global pancreas FF in patients without and with replacement myocardial fibrosis. In both graphs, the horizontal dotted line represents the upper limit of normal for global pancreas FF

The global pancreas FF showed a significant negative correlation with global heart T2* values (*R* =  − 0.249; *p* = 0.009) and a significant positive correlation with the number of segments with pathological T2* (*R* = 0.207; *p* < 0.0001). All 24 (7.8%) patients with significant MIO had pancreatic fatty replacement. A normal global pancreas FF showed a negative predictive value of 100% for MIO. Patients with significant MIO showed a significantly higher global pancreatic FF than patients without significant MIO [42.13 (27.36–55.88) % vs 24.37 (9.07–35.54%; *p* < 0.0001] (Fig. 3A).

### Pancreatic FF and cardiac function

No correlation was detected between global pancreatic FF and left (*R* =  − 0.021; *p* = 0.713) or right (*R* =  − 0.079; *p* = 0.179) ventricular ejection fractions.

The contrast medium was administered to 143 (46.4%) patients, and 56 (39.2%) of them showed replacement myocardial fibrosis. Only one patient showed an ischemic pattern. Global pancreatic FF was significantly higher in patients with myocardial fibrosis [27.73 (20.25–41.35) % vs 19.98 (7.38–29.73) %; *p* = 0.002] (Fig. 3B).

### Pancreatic FF and cardiac complications

**Fig. 4:**
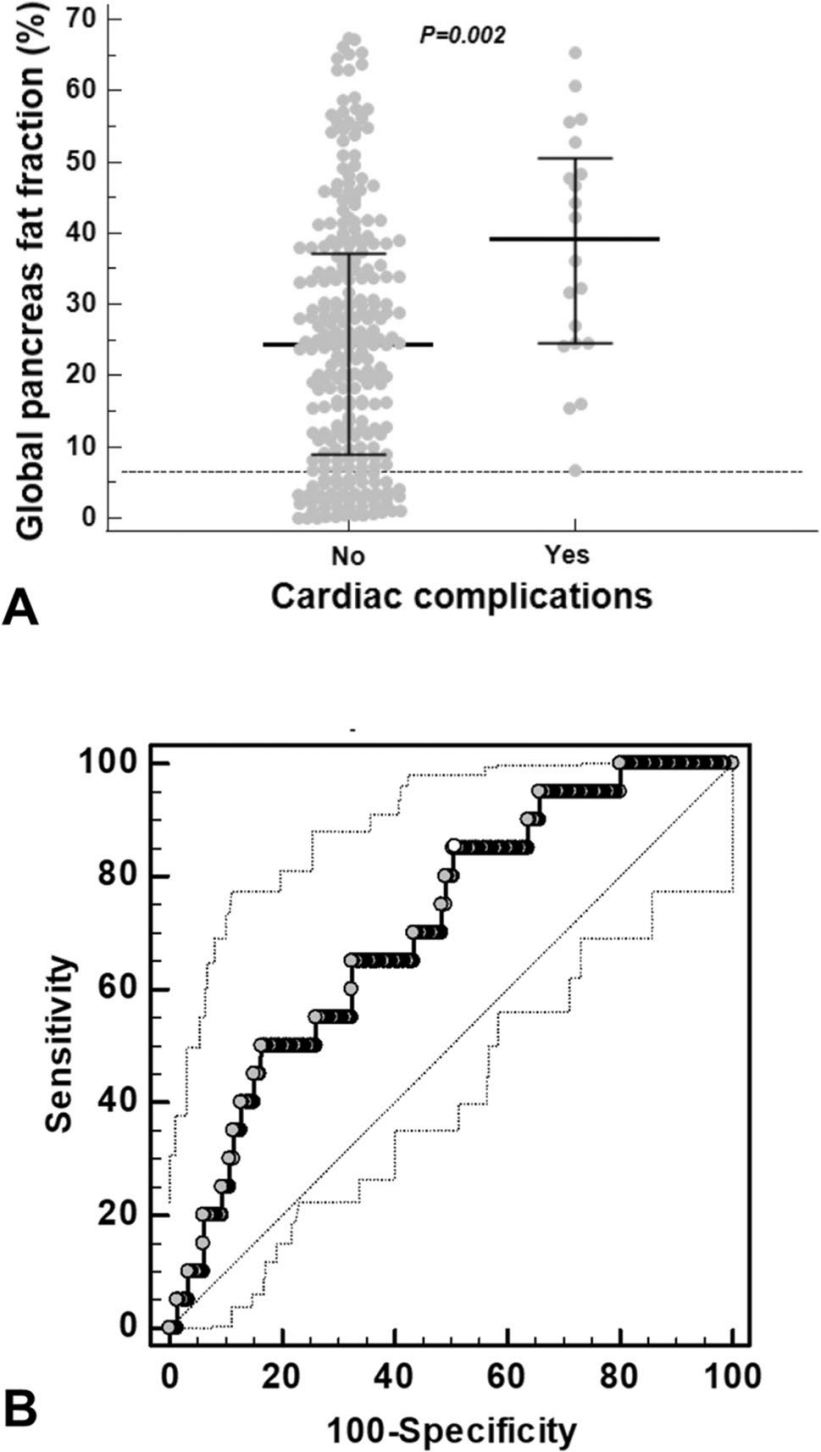
**A** Global pancreas FF in patients without and with cardiac complications. The horizontal dotted line represents the upper limit of normal for global pancreas FF. **B** ROC curve analysis of global pancreas FF to predict the presence of cardiac complications

Twenty patients had at least one cardiac complication: 5 HF, 10 arrhythmias (8 supraventricular and 2 ventricular), 3 HF + supraventricular arrhythmias, and 2 PH. Patients with cardiac complications showed a significantly higher global pancreas FF than patients free of complications [39.09 (24.56–50.55) % vs 24.37 (9.07–37.06) %; *p* = 0.002] (Fig. 4). No patient with cardiac complications had a normal global pancreas FF. At ROC curve analysis, a global pancreas FF > 24.20% predicted the presence of cardiac complications with a sensitivity = 85.0% and a specificity = 49.4% (*p* = 0.0002). The AUC was 0.71 (95% confidence interval = 0.65–0.76) (Fig. 4B).

## Discussion

The clinical significance of pancreatic fat replacement has gained considerable attention in recent years, and we explored the extent of pancreatic fatty replacement and its clinical correlates in well-treated TM patients.

The obtained upper limit of pancreatic FF is in close agreement with that one (6.2%) recommended by a recent meta-analysis including 9 studies, for a total of 1209 healthy individuals studied by MRI, although with different methods [46].

Eighty-two percent of our TM patients had a pancreatic fatty replacement, and this impressive prevalence is in line with the Pfifer’s study [18]. Moreover, as in the above-mentioned study, we did not detect significant differences among the regional pancreatic FF values.

We found a weak association between pancreatic fatty replacement and aging and we confirmed that fat accumulation could occur also in patients < 10 years of age [14]. As expected, patients with metabolic syndrome showed a significantly higher pancreatic FF. Our finding about the increased pancreatic FF among splenectomized TM patients could be explained by the fact that the spleen acts as storage for non-toxic iron [47] and lipids [48]. Midiri et al [9] did not find a correlation between pancreatic fatty replacement and age or history of splenectomy, likely due to the use of a semi-quantitative approach and the significantly smaller study population [9].

As previously shown in smaller cohorts of adults [18] and pediatric TM patients [14], pancreatic fatty replacement was correlated with pancreatic IO. All patients with fatty replacement had also pancreatic iron overload, confirming the hypothesis of the substitution of pancreatic cells, died because of the cytotoxic effects of iron, with adipose tissue [9]. A global pancreas T2* < 20.81 ms predicted the presence of fatty replacement with good sensitivity and specificity.

This is the first study showing an association between HCV infection and higher levels of fat accumulation in the pancreas. HCV can be present in human pancreatic β-cells [49] and it can lead to acinar cell apoptosis and irreversible replacement by adipocytes both directly [50] and indirectly through the increase in pancreatic iron load [8].

In non-diabetic patients, pancreatic FF was significantly associated with plasma glucose levels but not with HOMA-IR. In TM patients, insulin resistance is caused by hepatic IO that interferes with the suppression of hepatic glucose production from insulin, and by iron deposition in the muscle that decreases the glucose uptake [6], while a reduced insulin secretion can be present also in normoglycemic patients [51]. Moreover, the use of indices based on fasting glucose and insulin concentrations has its limitations [38]. Patients with normal glucose metabolism showed a significantly lower global pancreas FF than patients with IFG, IGT, and DM; a normal global pancreas FF value showed a negative predictive value of 100% for disturbances of glucose metabolism. We introduced a cutoff of 15% for the prediction of abnormal glucose metabolism, which matches well with the cutoff of 18% found in TM pediatric patients for the discrimination between IFG and normal glucose function [14]. The low specificity of our cutoff can be the consequence of a latency time between the fatty replacement of pancreatic parenchyma and the overt disease. Pancreatic FF resulted superior to pancreatic iron in discriminating patients with altered glucose metabolism.

This is the first study demonstrating a significant association between MIO and pancreatic FF and revealing that, in addition to normal global pancreas T2* values [7, 8, 52], also a normal FF had a 100% negative predictive value for significant MIO.

Pancreatic FF was not associated with cardiac function, which can be impaired by different causes besides iron [34], but it was increased in patients with replacement myocardial fibrosis. This finding further reinforces the crucial role of HCV infection in cardiac impairment, by causing myocardial fibrosis both directly [35, 53] and indirectly through the development of DM [20, 54]. As myocardial fibrosis was shown to be the strongest CMR predictor and DM the strongest clinical predictor for HF and cardiac complications in TM [36], it is not surprising, although never demonstrated before, that pancreatic fatty replacement was associated with the development of cardiac complications.

### Limitations

We did not perform MR spectroscopy, which is generally considered the gold standard for non-invasive fat and metabolite quantification [46].

Due to its high risk, no histological confirmation was performed for iron and fat content measurements by MRI.

## Conclusions

In TM, pancreatic fatty replacement is a frequent clinical entity, predicted by a pancreatic T2* < 20.81 ms and associated with the risk of developing alterations in glucose metabolism. Pancreatic fatty replacement is a strong risk marker for cardiac iron, replacement fibrosis, and complications, highlighting a deep connection between pancreatic and cardiac impairment. Since pancreatic FF can be easily obtained by the same T2* sequence employed for iron overload assessment, it should be included in the routine MRI assessment of TM patients for early identification of patients at high risk for glucose dysregulation and cardiac damage.

## Abbreviations

AUC: Area under the curve
DM: Diabetes mellitus
E-MIOT: Extension-Myocardial Iron Overload in Thalassemia
FF: Fat fractions
FPG: Fasting plasma glucose
HF: Heart failure
HOMA-IR: Omeostasis model assessment of insulin resistance
IFG: Impaired fasting glucose
IGT: Impaired glucose tolerance
LGE: Late gadolinium enhancement
LIC: Liver iron concentration
LV: Left ventricle
MIO: Myocardial iron overload
MRI: Magnetic resonance imaging
NGT: Normal glucose tolerance
OGTT: Oral glucose tolerance test
PH: Pulmonary hypertension
PSI: Pancreatic signal index
ROC: Receiver-operating characteristic
ROI: Regions of interest
SD: Standard deviation
SIR: Signal intensity ratio
TM: Thalassemia major

## Declarations

### Guarantor

The scientific guarantor of this publication is Alessia Pepe.

### Statistics and biometry

One of the authors has significant statistical expertise.

### Study subjects or cohorts overlap

Some study subjects have been previously reported in other studies of the E-MIOT project.

### Methodology

• retrospective

• observational

• multicenter study

## References

[1] Ladis V, Chouliaras G, Berdousi H, Kanavakis E, Kattamis C (2005). Longitudinal study of survival and causes of death in patients with thalassemia major in Greece. Ann N Y Acad Sci.

[2] Modell B, Khan M, Darlison M, Westwood MA, Ingram D, Pennell DJ (2008). Improved survival of thalassaemia major in the UK and relation to T2* cardiovascular magnetic resonance. J Cardiovasc Magn Reson.

[3] Pepe A, Pistoia L, Gamberini MR (2022). National networking in rare diseases and reduction of cardiac burden in thalassemia major. Eur Heart J.

[4] Mordarska K, Godziejewska-Zawada M (2017) Diabetes in the elderly. Prz Menopauzalny 16:38-43 10.5114/pm.2017.68589 PMC5509969 28721127

[5] Cooksey RC, Jouihan HA, Ajioka RS (2004). Oxidative stress, beta-cell apoptosis, and decreased insulin secretory capacity in mouse models of hemochromatosis. Endocrinology.

[6] Merkel PA, Simonson DC, Amiel SA (1988). Insulin resistance and hyperinsulinemia in patients with thalassemia major treated by hypertransfusion. N Engl J Med.

[7] Noetzli LJ, Papudesi J, Coates TD, Wood JC (2009). Pancreatic iron loading predicts cardiac iron loading in thalassemia major. Blood.

[8] Pepe A, Pistoia L, Gamberini MR (2020). The close link of pancreatic iron with glucose metabolism and with cardiac complications in thalassemia major: a large, multicenter observational study. Diabetes Care.

[9] Midiri M, Lo Casto A, Sparacia G (1999). MR imaging of pancreatic changes in patients with transfusion-dependent beta-thalassemia major. AJR Am J Roentgenol.

[10] Reeder SB, Sirlin CB (2010). Quantification of liver fat with magnetic resonance imaging. Magn Reson Imaging Clin N Am.

[11] Pezzilli R, Calculli L (2014). Pancreatic steatosis: is it related to either obesity or diabetes mellitus?. World J Diabetes.

[12] Papakonstantinou O, Ladis V, Kostaridou S (2007). The pancreas in beta-thalassemia major: MR imaging features and correlation with iron stores and glucose disturbances. Eur Radiol.

[13] Hines CD, Yu H, Shimakawa A, McKenzie CA, Brittain JH, Reeder SB (2009). T1 independent, T2* corrected MRI with accurate spectral modeling for quantification of fat: validation in a fat-water-SPIO phantom. J Magn Reson Imaging.

[14] Huang J, Shen J, Yang Q (2021). Quantification of pancreatic iron overload and fat infiltration and their correlation with glucose disturbance in pediatric thalassemia major patients. Quant Imaging Med Surg.

[15] Meloni A, De Marchi D, Positano V (2015). Accurate estimate of pancreatic T2* values: how to deal with fat infiltration. Abdom Imaging.

[16] Hernando D, Kramer JH, Reeder SB (2013). Multipeak fat-corrected complex R2* relaxometry: theory, optimization, and clinical validation. Magn Reson Med.

[17] Hernando D, Levin YS, Sirlin CB, Reeder SB (2014). Quantification of liver iron with MRI: state of the art and remaining challenges. J Magn Reson Imaging.

[18] Pfeifer CD, Schoennagel BP, Grosse R (2015). Pancreatic iron and fat assessment by MRI-R2* in patients with iron overload diseases. J Magn Reson Imaging.

[19] Santarelli MF, Meloni A, De Marchi D (2018). Estimation of pancreatic R2* for iron overload assessment in the presence of fat: a comparison of different approaches. MAGMA.

[20] Pepe A, Meloni A, Rossi G (2013). Cardiac complications and diabetes in thalassaemia major: a large historical multicentre study. Br J Haematol.

[21] Pepe A, Positano V, Santarelli F (2006). Multislice multiecho T2* cardiovascular magnetic resonance for detection of the heterogeneous distribution of myocardial iron overload. J Magn Reson Imaging.

[22] Ramazzotti A, Pepe A, Positano V (2009). Multicenter validation of the magnetic resonance t2* technique for segmental and global quantification of myocardial iron. J Magn Reson Imaging.

[23] Meloni A, De Marchi D, Pistoia L (2019). Multicenter validation of the magnetic resonance T2* technique for quantification of pancreatic iron. Eur Radiol.

[24] Restaino G, Meloni A, Positano V (2011). Regional and global pancreatic T*(2) MRI for iron overload assessment in a large cohort of healthy subjects: normal values and correlation with age and gender. Magn Reson Med.

[25] Positano V, Salani B, Pepe A (2009). Improved T2* assessment in liver iron overload by magnetic resonance imaging. Magn Reson Imaging.

[26] Meloni A, Positano V, Pepe A (2010). Preferential patterns of myocardial iron overload by multislice multiecho T*2 CMR in thalassemia major patients. Magn Reson Med.

[27] Positano V, Pepe A, Santarelli MF (2007). Standardized T2* map of normal human heart in vivo to correct T2* segmental artefacts. NMR Biomed.

[28] Meloni A, Luciani A, Positano V (2011). Single region of interest versus multislice T2* MRI approach for the quantification of hepatic iron overload. J Magn Reson Imaging.

[29] Wood JC, Enriquez C, Ghugre N (2005). MRI R2 and R2* mapping accurately estimates hepatic iron concentration in transfusion-dependent thalassemia and sickle cell disease patients. Blood.

[30] Meloni A, Rienhoff HY, Jones A, Pepe A, Lombardi M, Wood JC (2013). The use of appropriate calibration curves corrects for systematic differences in liver R2* values measured using different software packages. Br J Haematol.

[31] Cerqueira MD, Weissman NJ, Dilsizian V (2002). Standardized myocardial segmentation and nomenclature for tomographic imaging of the heart: a statement for healthcare professionals from the Cardiac Imaging Committee of the Council on Clinical Cardiology of the American Heart Association. Circulation.

[32] He T, Gatehouse PD, Smith GC, Mohiaddin RH, Pennell DJ, Firmin DN (2008). Myocardial T2* measurements in iron-overloaded thalassemia: an in vivo study to investigate optimal methods of quantification. Magn Reson Med.

[33] Meloni A, Righi R, Missere M (2021). Biventricular reference values by body surface area, age, and gender in a large cohort of well-treated thalassemia major patients without heart damage using a multiparametric CMR approach. J Magn Reson Imaging.

[34] Marsella M, Borgna-Pignatti C, Meloni A (2011). Cardiac iron and cardiac disease in males and females with transfusion-dependent thalassemia major: a T2* magnetic resonance imaging study. Haematologica.

[35] Pepe A, Meloni A, Borsellino Z (2015). Myocardial fibrosis by late gadolinium enhancement cardiac magnetic resonance and hepatitis C virus infection in thalassemia major patients. J Cardiovasc Med (Hagerstown).

[36] Pepe A, Meloni A, Rossi G (2018). Prediction of cardiac complications for thalassemia major in the widespread cardiac magnetic resonance era: a prospective multicentre study by a multi-parametric approach. Eur Heart J Cardiovasc Imaging.

[37] Pepe A, Positano V, Capra M (2009). Myocardial scarring by delayed enhancement cardiovascular magnetic resonance in thalassaemia major. Heart.

[38] Matthews DR, Hosker JP, Rudenski AS, Naylor BA, Treacher DF, Turner RC (1985). Homeostasis model assessment: insulin resistance and beta-cell function from fasting plasma glucose and insulin concentrations in man. Diabetologia.

[39] Angelucci E, Brittenham GM, McLaren CE (2000). Hepatic iron concentration and total body iron stores in thalassemia major. N Engl J Med.

[40] Meloni A, Maggio A, Positano V (2020). CMR for myocardial iron overload quantification: calibration curve from the MIOT network. Eur Radiol.

[41] De Sanctis V, Soliman AT, Elsedfy H (2016). The ICET-A recommendations for the diagnosis and management of disturbances of glucose homeostasis in thalassemia major patients. Mediterr J Hematol Infect Dis.

[42] Alberti KG, Eckel RH, Grundy SM (2009). Harmonizing the metabolic syndrome: a joint interim statement of the International Diabetes Federation Task Force on Epidemiology and Prevention; National Heart, Lung, and Blood Institute; American Heart Association; World Heart Federation; International Atherosclerosis Society; and International Association for the Study of Obesity. Circulation.

[43] Jessup M, Abraham WT, Casey DE (2009). 2009 focused update: ACCF/AHA guidelines for the diagnosis and management of heart failure in adults: a report of the American College of Cardiology Foundation/American Heart Association Task Force on Practice Guidelines: developed in collaboration with the International Society for Heart and Lung Transplantation. Circulation.

[44] Buxton AE, Calkins H, Callans DJ (2006). ACC/AHA/HRS 2006 key data elements and definitions for electrophysiological studies and procedures: a report of the American College of Cardiology/American Heart Association Task Force on Clinical Data Standards (ACC/AHA/HRS Writing Committee to Develop Data Standards on Electrophysiology). Circulation.

[45] Cogliandro T, Derchi G, Mancuso L (2008). Guideline recommendations for heart complications in thalassemia major. J Cardiovasc Med (Hagerstown).

[46] Singh RG, Yoon HD, Wu LM, Lu J, Plank LD, Petrov MS (2017). Ectopic fat accumulation in the pancreas and its clinical relevance: a systematic review, meta-analysis, and meta-regression. Metabolism.

[47] Brewer CJ, Coates TD, Wood JC (2009). Spleen R2 and R2* in iron-overloaded patients with sickle cell disease and thalassemia major. J Magn Reson Imaging.

[48] Schmidt HH, Wagner S, Manns M (1997). The spleen as a storage pool in lipid metabolism. Am J Gastroenterol.

[49] Masini M, Campani D, Boggi U (2005). Hepatitis C virus infection and human pancreatic beta-cell dysfunction. Diabetes Care.

[50] Smits MM, van Geenen EJ (2011). The clinical significance of pancreatic steatosis. Nat Rev Gastroenterol Hepatol.

[51] Cario H, Holl RW, Debatin KM, Kohne E (2003). Insulin sensitivity and beta-cell secretion in thalassaemia major with secondary haemochromatosis: assessment by oral glucose tolerance test. Eur J Pediatr.

[52] Meloni A, Restaino G, Missere M (2015). Pancreatic iron overload by T2* MRI in a large cohort of well treated thalassemia major patients: can it tell us heart iron distribution and function?. Am J Hematol.

[53] Omura T, Yoshiyama M, Hayashi T (2005). Core protein of hepatitis C virus induces cardiomyopathy. Circ Res.

[54] Vassalle C, Petta S, Pepe A, Craxi A, Bondin M, Cacoub P (2018). Expert opinion on managing chronic HCV in patients with cardiovascular disease. Antivir Ther.

